# Characterization of Novel Partially Bio-Based, Waste-Derived Composites for Thermal and Acoustic Performance in Buildings

**DOI:** 10.3390/polym18111401

**Published:** 2026-06-04

**Authors:** Mohamed Ali, Redhwan Almuzaiqer, Hassan Alshehri, Mohammed A. Alanazi, Turki Almudhhi, Abdullah Nuhait

**Affiliations:** Mechanical Engineering Department, College of Engineering, King Saud University, P.O. Box 800, Riyadh 11421, Saudi Arabiahashehri@ksu.edu.sa (H.A.); malanazif@ksu.edu.sa (M.A.A.); anuhait@ksu.edu.sa (A.N.)

**Keywords:** discarded medical isolation gowns, wasted coffee filter papers, recycling waste materials, thermal insulation materials, sound absorption materials, thermal stability analysis

## Abstract

New partially bio-based, waste-derived composites are manufactured from date palm surface fibers (DPSF), waste coffee filters (CFP), and disposable medical isolation gowns (MIG). These three disposable raw materials fill landfills and create an environmental problem. Therefore, the objective of this current study is to use such materials in creating promised thermal insulation and sound absorption boards. Six hybrid composites with different compositions were made using Polyvinyl acetate (PVA) wood adhesive as a binder. Three of them were made of DPSF and MIG, and the other three were composed of DPSF and the CFP. Different tests were performed on the developed composites, such as thermal conductivity measurements, sound absorption and noise reduction determination, surface morphology image analysis, thermogravimetric analysis, and three-point bending tests. The results showed that the thermal conductivity coefficients for the hybrids DPSF + MIG and DPSF + CFP are in the ranges 0.0493–0.0613 W/(m·K) and 0.052–0.065 W/(m·K), respectively, over the temperature range 24–82 °C. The sound absorption coefficient (SAC) is greater than 0.4 for all composites at frequency bands greater than 500 Hz. The noise reduction coefficient (NRC) is ≥0.45 for all composites. Surface morphology images of the composites were also reported. The results also show that the composites are thermally stable at temperatures up to 258.3 °C. The flexural modulus ranges between 5.0 and 8.46 MPa for the medical isolation gown composites and 2.49 and 5.57 MPa for the coffee filter paper composites. The hybrid composites have a lower moisture content of 0.51% to 2.5%. These promising results support the use of these composites for thermal insulation and sound absorption in building construction as alternatives to conventional thermal insulations derived from crude fuels.

## 1. Introduction

One of the crucial aspects of environmental sustainability requires applying the circular economy basis. Every day, about eight million tons of coffee are consumed [1]. The Saudi Arabia coffee filter paper market was valued at approximately USD 21.13 million in 2024. This entire amount is effectively “wasted” in the sense of being a single-use product that enters the waste stream [2]. Another similar statistic from the International Coffee Organization showed that global coffee consumption reached 166.34 million 60 kg bags in the 2019–2021 period [3]. This massive consumption produces tremendous single-use coffee filter papers (CFP) as waste. These filters cannot be recycled as paper waste because they have trapped oil in their material [4]. Furthermore, the economic value of disposable medical isolation gowns encompasses a global market projected to reach over USD 8.5 billion by 2030, driven by factors like infection control and operational efficiency. This value is defined by direct market worth, significant cost savings from averted infections, and logistical benefits [5]. Saudi Arabia’s hospital gown market, including both disposable and reusable types, was valued at approximately USD 135.12 million in 2024. This market is projected to grow, reaching an estimated USD 237.95 million by 2030 [6]. Saudi Arabia has an area of 1073 km^2^ cultivated land with 28.5 million date palm trees [7] and produces eleven million tons of agricultural waste annually, the majority of which belongs to date palm trees. Economic analyses showed that valuable benefits could be obtained from this waste [8,9]. However, if that waste is burned in the air, as commonly happens in some areas worldwide, it causes pollution and increases the negative environmental impact [10]. Rizkiansyah et al. [11] utilized coffee pulp waste to produce coffee filter papers, thereby reducing waste accumulation and its environmental impact. Rendón et al. [12] investigated the impact of CFP size, color, and origin on diterpene concentrations in filter coffee brews. Wasted paper pulp and coffee grounds were used to develop composite materials, which were then used to produce seedling pots [13]. Boughanmi et al. [14] presented a comprehensive review on transforming spent coffee grounds into composite materials for waste valorization. To reduce the large amount of synthetic isolation gowns that are wasted and end up in landfills, creating pollution and environmental hazards, Scott et al. [15] developed a new degradable textile suitable material from which to create compostable isolation gowns that meet healthcare standards. Woven fabric waste and textile waste from the textile industry were used to develop thermal insulation for buildings by Briga-Sá et al. [16]. The thermal conductivity coefficient of their products was found to be comparable to that of expanded polystyrene, extruded polystyrene, and mineral wool. Recently, Ali et al. [17] developed thermal insulation and sound-absorbing materials from discarded medical isolation gowns and waste coffee filters as raw materials. Their study showed that the thermal conductivity of the boards and their hybrid ones were in the range 0.038 to 0.070 W/(m·K), the sound absorption coefficient was greater than 0.4 for all boards at frequencies above 500 Hz, and the noise reduction coefficient was greater than 0.4. Ali and Alabdulkarem [18] used waste date palm surface fibers to produce thermal insulation materials for building applications using cornstarch as a binder. The thermal conductivity coefficient of their produced materials was in the range of 0.0475–0.0697 W/(m.K). Alabdulkarem et al. [19] developed hybrid thermal insulation and sound absorption materials made from date palm surface fibers, agave fibers, and apple of Sodom fibers using different binders. Their hybrid prototypes have a thermal conductivity in the range of 0.04234–0.05291 W/(m·K) and a high sound absorption coefficient at the middle frequencies. Other hybrid thermal insulation and sound absorption materials were reported by Ali et al. [20], who used waste date palm surface fibers and discarded pineapple leaf fibers. In another publication by Ali et al. [21], hybrid materials were made from date palm surface fibers and waste tea bags. The average thermal conductivity of the hybrid in Ref. [20] was 0.054–0.07 W/(m·K), and the sound absorption coefficient was greater than 0.5 for frequencies above 1000 Hz. In addition, hybrid samples in Ref. [21] had a thermal conductivity less than 0.07 W/(m·K) at 24 °C, and the noise reduction coefficient was greater than 0.37 using the average one-third octave of the sound absorption coefficients at frequencies 250, 500, 1000, and 2000 Hz.

Therefore, this study presents a novel solution using date palm surface fibers (DPSF) hybridized with both coffee filter papers (CFP) and medical isolation gowns (MIGs) to produce thermal insulation and sound absorption materials to lower the environmental impact. It should be clarified that although the reinforcing phases of the developed composites are derived from renewable (DPSF) or recycled (CFP, MIG) streams, the polyvinyl acetate (PVA) binder is a synthetic, petroleum-derived polymer. Following the definition adopted in ISO 16620-1:2015 [22], the materials are therefore more accurately described as “partially bio-based, waste-derived composites” rather than full bio-composites.

## 2. Materials and Methods

### 2.1. Materials

**Figure 1:**
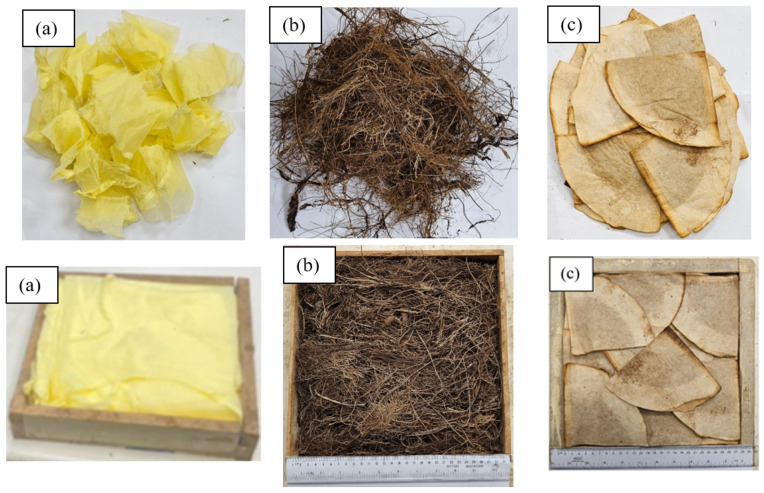
The three waste raw materials used in this study. The **upper** ones present the loose form and the **lower** ones present the box compact form of size 30 × 30 cm^2^: (**a**) MIG, (**b**) DPSF, and (**c**) CFP.

Regarding coffee filter papers (CFP), raw waste materials were collected from cafés. Date palm surface fibers (DPSF) were obtained from the agricultural authority during palm tree trimming. Discarded medical isolation gowns (MIGs) were simulated using new ones. The coffee filters (CFP) and date palm surface fibers (DPSF) were washed with water to remove any coffee residue or dust, respectively. Following Ref. [23], the samples were dried in an oven at 80 °C for 12 h to prevent hornification. This process preserves inter-fiber porosity, which is essential for reducing thermal conductivity and enhancing sound absorption. Additionally, this moderate temperature prevents surface yellowing, fiber embrittlement, and thermal degradation of polypropylene-based MIGs. Figure 1 shows the dry waste raw materials in both loose and compacted forms, prepared for thermal conductivity measurement using a heat flow meter (HFM 436 Lambda, NETZSCH-Gerätebau GmbH Wittelsbacherstraße 42, 95100 Selb, Germany).

### 2.2. Methods

**Figure 2:**
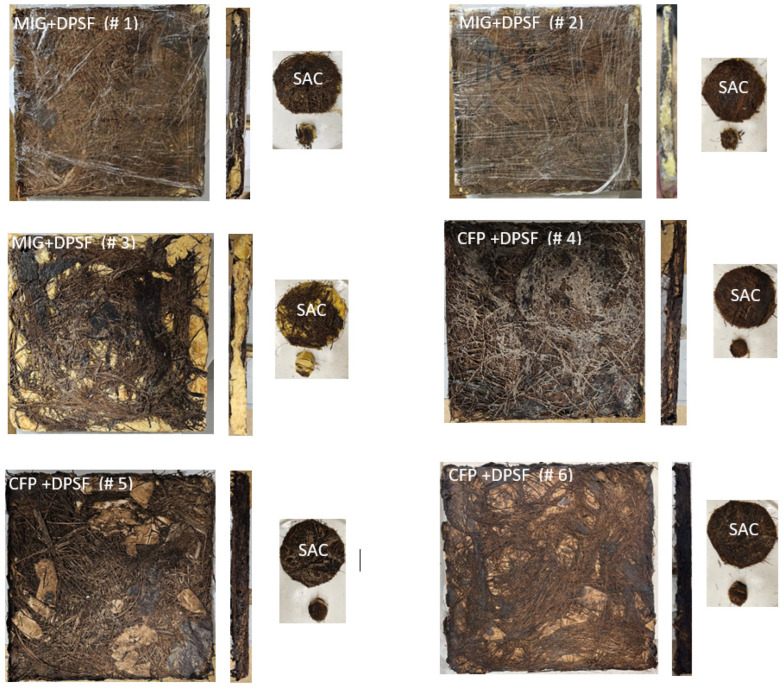
Hybrid composite samples with different MIG/DPSF (samples (#1–#3)) and CFP/DPSF (samples (#4–#6)) compositions were produced as specified in Table 1. Samples designated “SAC” were used for sound absorption coefficient measurements.

Hybrid composites were prepared with varying compositions using either DPSF and CFP or DPSF and MIG. Polyvinyl acetate wood adhesive (PVA, C_4_H_6_O_2_, molar mass per unit = 86.09 g/mol or the typical weight-average molecular weight of the dispersed PVA in the range commonly reported for wood-grade adhesives (approximately 1.0 × 10^5^ to 1.5 × 10^5^ g.mol^−1^)) was used as a binder, with its properties detailed in Ref. [24]. The fabrication process began with the preparation of a 3:1 water-to-resin solution, into which the raw materials were fully immersed. For the DPSF/CFP composite, CFP was placed between layers of DPSF in a 30 × 30 cm^2^ stainless steel mold. This assembly was then subjected to a mechanical press to achieve a specific thickness (γ) before being dried in a convection oven for 72 h at 80 °C. A similar procedure was followed to create the DPSF/MIG hybrid composite. The mold was lined with an oiled aluminum sheet to simplify the extraction of the finished sample. A summary of the composite compositions, specifications, and dimensions is provided in Table 1, and the finished composites are shown in Figure 2. Although the photographs in Figure 2 show a visually heterogeneous surface, this heterogeneity is expected and inherent in multi-component fibrous waste-based composites. Reproducibility can be nearly achieved by (i) using the same standard fiber size, (ii) keeping the dried mass of each polymer constant, (iii) using a single mold geometry (30 × 30 cm^2^), and (iv) applying an identical pressing-and-drying protocol to every sample. However, the polymerized binder ratio in the final dried composites is the only parameter that cannot be controlled on a lab scale.

#### 2.2.1. Composite Formulation Design

Six hybrid formulations were retained for full characterization (samples #1–#6, Table 1): three DPSF + MIG composites and three DPSF + CFP composites. The initial matrix of the experimental dry mass fraction of DPSF, MIG, and CFP is shown in Table 2. Composite dry mixtures were decided, such that they cover a wide range of mass fractions in the range of 25% to 75% on a dry basis (no binder) of each component. This range was chosen so that the influence of composition on thermal conductivity, sound absorption, mechanical resistance, and density could be evaluated systematically rather than at a single design point. However, after preparing the binder solution as mentioned earlier in Section 2.2, mixing it with the dry hybrid composites shown in Table 2, and then pressing and drying, the sample total mass was obtained (including the binder) and the new actual mass fraction of each constituent was calculated, as shown in Table 1.

#### 2.2.2. Thermal Conductivity (α) Determination

The thermal conductivity of the produced composites (Figure 2) was measured using a Lambda HFM 436 heat flow meter (bench-type) apparatus (Netzsch, Selb, Germany) [25]. This heat flow meter is a calibrated instrument that tests according to the ASTM method [26]. The HFM uses samples measuring 30 × 30 × γ cm^3^, with the thickness (γ) for each sample listed in Table 1. Measurements were conducted over a temperature range of 10 °C to 80 °C. According to the manufacturer, the accuracy of the thermal conductivity measurement is ±1.0–3.0% W/(m·K), and the temperature accuracy is ±0.01 °C. It should be noted that this HFM is specified for measuring the thermal conductivity coefficient for insulation materials in the range of 0.005 to 0.50 W/(m·K), as specified by the manufacturer [25].

#### 2.2.3. Sound Absorption Measurements (SA)

The sound absorption coefficient (SAC) was measured for all composite samples listed in Table 1. Impedance tubes with diameters of 10 cm and 3 cm were used. The composite samples used for this test are shown in Figure 2, labeled SAC. Using both tubes allows for the measurement of the SAC across a frequency range of 100 Hz to 6000 Hz. The impedance tube operation principle, microphone setup, and the frequency range of each tube are detailed in Ali et al. [27]. The SAC test conforms to both ISO 10534-1 [28] and ISO 10534-2 [29] standards.

#### 2.2.4. Surface Morphology of the Composites

The surface morphology of the composite sample number 2 (MIG + DPSF) and number 5 (CFP + DPSF) was determined using Scanning Electron Microscopy (JEOL, Freising, Germany; JSM7600F) at different magnifications.

#### 2.2.5. Thermogravimetric Analysis

The thermal stability analysis of composite samples 2 (MIG + DPSF) and 5 (CFP + DPSF) was performed using a thermogravimetric instrument provided by TA Instruments (SDT Q600 V20.9 Build 20 setup, Waters Corporation, New Castle, DE 19720,USA). This instrument is equipped with a nitrogen purge gas. The test was conducted up to 800 °C at a rate of 10 °C/min.

#### 2.2.6. Flexure Test

The three-point flexure test is used to determine the flexural modulus E_*f*_, flexural stress *σ_f_*, and flexural strain *ϵ_f_* for all composite samples. Table 3 presents the dimensions of the specimens used.

**Figure 3:**
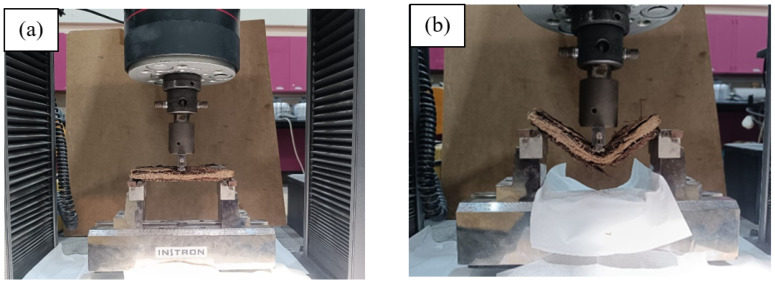
The universal testing machine (**a**) before bending and (**b**) after bending.

The universal testing machine (UTM, INSTRON 5984, Instron, Norwood, MA, USA) (Figure 3) was used to record the different physical parameters. This machine has a crosshead speed of 2 mm/min.

#### 2.2.7. Moisture Test

**Figure 4:**
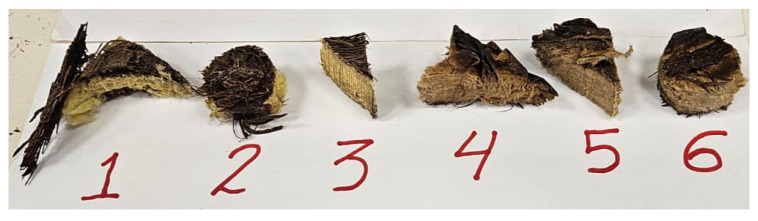
Specimen of the composites used for moisture content test. Numbers refer to sample numbers as appear in Table 1.

Small pieces of the studied composites (Figure 4) were dried in a convection oven at 80 °C for 10 h. The dried mass from the oven was scaled and assigned *m*2. The mass of each piece was traced at the laboratory ambient condition of relative humidity 51.7% and temperature 21.6 °C. The traced mass was recorded every 5 min and assigned *m*1. The moisture content was estimated using Equation (1), following the ASTM D2974-07A [30] standard. The moisture mass percentage for each sample composite was graphed against time until a steady state was achieved.

(1) $$\%\;\mathrm{of}\;\mathrm{moisture}\;\mathrm{content}=\frac{m1-m2}{m2}\times 100$$

## 3. Uncertainty Analyses

The Taylor [31] propagation method for uncertainty analysis is used to determine the uncertainty in flexural property, acoustic parameters, and thermal conductivity measurements, where the function *f* depends on the independent variables *x*, *y*, *z*, …, and the combined uncertainty is given by:

$$\Delta f\;=\;\sqrt{{\left(\frac{\partial f}{\partial x}\;\cdot \;\Delta x\right)}^{2}\;+\;{\left(\frac{\partial f}{\partial y}\;\cdot \;\Delta y\right)}^{2}\;+\;\cdots }$$

### 3.1. Flexural Property

The following equations are used to determine the uncertainty in the flexural stress $\sigma_{f}=\frac{3FL}{2bd^{2}}$, flexural strain $\varepsilon_{f}=\frac{6Dd}{L^{2}}$, and flexural modulus $E_{f}=\frac{L^{3}S}{4bd^{3}}$, where *F*, *S*, and *D* stand for the applied load, slope of the linear elastic region of the load/deflection curve (N/mm), and the deflection, respectively.

$$\Delta \sigma_{f}=\sqrt{{\left(\frac{\partial \sigma_{f}}{\partial F}\;\Delta F\right)}^{2}+{\left(\frac{\partial \sigma_{f}}{\partial L}\;\Delta L\right)}^{2}+{\left(\frac{\partial \sigma_{f}}{\partial b}\;\Delta b\right)}^{2}+{\left(\frac{\partial \sigma_{f}}{\partial d}\;\Delta d\right)}^{2}}$$

$$\Delta \varepsilon_{f}=\sqrt{{\left(\frac{\partial \varepsilon_{f}}{\partial D}\;\Delta D\right)}^{2}+{\left(\frac{\partial \varepsilon_{f}}{\partial d}\;\Delta d\right)}^{2}+{\left(\frac{\partial \varepsilon_{f}}{\partial L}\;\Delta L\right)}^{2}}$$

$$\Delta E_{f}=\sqrt{{\left(\frac{\partial E_{f}}{\partial L}\;\Delta L\right)}^{2}+{\left(\frac{\partial E_{f}}{\partial S}\;\Delta S\right)}^{2}+{\left(\frac{\partial E_{f}}{\partial b}\;\Delta b\right)}^{2}+{\left(\frac{\partial E_{f}}{\partial d}\;\Delta d\right)}^{2}}$$

Table 4 summarizes the uncertainty of the flexural properties for all specimens.

### 3.2. Acoustic Uncertainty

Applying the propagation of uncertainty formula to the noise reduction coefficient (NRC) equation, we obtain the following:

Δ (NRC) = (1/4) · √[(ΔSAC_250_)^2^ + (ΔSAC_500_)^2^ + (ΔSAC_1000_)^2^ + (ΔSAC_2000_)^2^]

Assuming the uncertainty in the SAC measurement (Δ SAC) is constant across the four frequencies, the equation simplifies to:

Δ (NRC) = (1/4) · √[4 · (Δ SAC)^2^] = (1/4) · 2 · Δ SAC = Δ SAC/2

Based on a comprehensive review of the literature on impedance tube round-robin tests and uncertainty analyses (Schultz et al. [32]), a conservative value for the uncertainty in a single SAC measurement is adopted, Δ SAC = ±0.05, using the simplified formula and the given uncertainty value for SAC: Δ (NRC) = 0.05/2 = ±0.025. This means the uncertainty for the NRC value is constant for all specimens, as it depends only on the assumed uncertainty of the SAC measurement itself. Table 5 summarizes the measured NRC values and their calculated absolute and relative uncertainties for all specimens. The absolute uncertainty Δ (NRC) is identical for all samples (±0.025), whereas the relative uncertainty depends on the magnitude of the NRC value itself.

### 3.3. Thermal Conductivity

Based on the uncertainty values provided by the manufacturer of the heat flow meter (NETZSCH HFM 436), Table 6 presents the uncertainty of the thermal conductivity for all composites at the reference temperatures of 25 °C and 80 °C.

## 4. Results and Discussion

**Figure 5:**
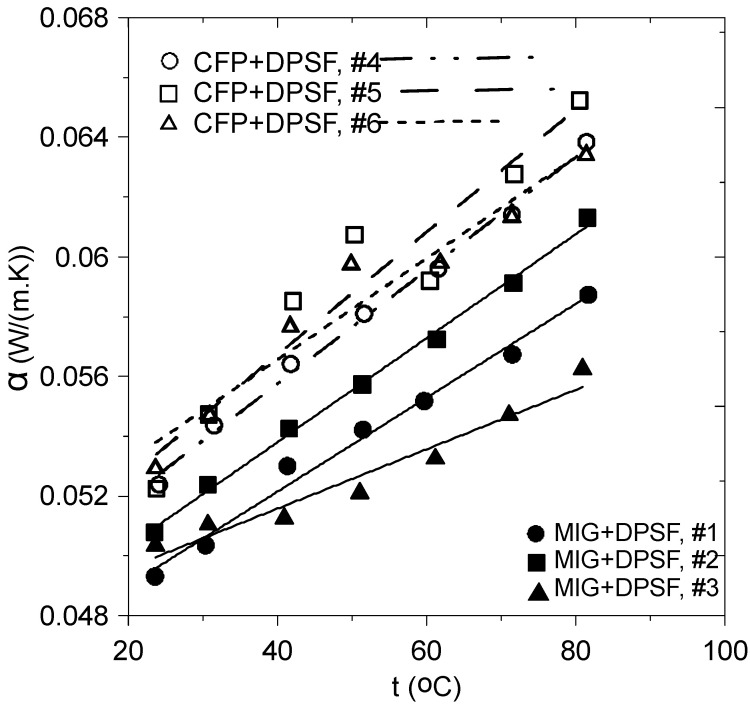
Thermal conductivity coefficient profiles for all composites, with curve-fitting lines connecting the points.

Figure 5 displays the thermal conductivity coefficient (α) for all composite samples listed in Table 1. Outline (hollow) and solid symbols represent hybrid composites made of DPSF with both CFP and MIG, respectively. The minimum and maximum α values for MIG and DPSF hybrid composites are 0.0493 and 0.0613 W/(m·K), occurring at temperatures of 23.6 and 81.6 °C, respectively. Consequently, for this temperature range, α increases by only 0.0094, 0.0105, and 0.0059 W/(m·K) for composite samples 1, 2, and 3, respectively. This increase indeed indicates that α is a weak function of temperature in this range. For the same temperature range, the other composite samples made of CFP and DPSF show increases in α of 0.0114, 0.0130, and 0.0105 W/(m·K) for samples 4, 5, and 6, respectively. Furthermore, the minimum and maximum α for these composites are 0.052 and 0.065 W/(m·K), respectively. Therefore, these results give promise for using these composites as an alternative source of thermal insulation instead of conventional synthetic and petrochemical thermal insulation materials derived from crude oils. The linear fitting correlation for the data points is obtained as follows:

α = A t + B(2)

where t is the temperature and A and B are constants given in Table 7, with the coefficient of determination R^2^ corresponding to each composite.

Table 8 presents a comparison between the conventional and unconventional thermal conductivity coefficient of the insulation materials cited in the literature and those of the current hybrid composites. This comparison demonstrates that the newly developed hybrid composites perform similarly to traditional petroleum-based thermal insulation. This underscores the potential of these composites to improve environmental sustainability in building insulation. Furthermore, the thermal conductivity coefficients of these composites are within the same range as those of similar unconventional materials. However, caution is required when making these comparisons, as the materials differ in composition and structure, preventing a direct density-to-density evaluation.

**Figure 6:**
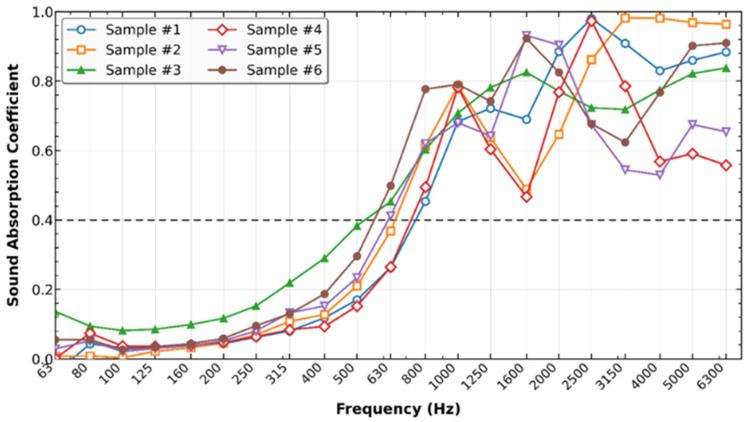
Composites’ SAC profiles per 1/3 octave band.

Figure 6 presents the sound absorption coefficient (SAC) per one-third octave band for all composite samples. The SAC exceeds 0.4 for samples 1, 2, 3, 4, 5, and 6 at frequency bands greater than or equal to 800, 630, 500, 800, 630, and 565 Hz, respectively. This observation indicates that these composites have practical value for sound absorption applications, as classified by Ref. [38].

**Figure 7:**
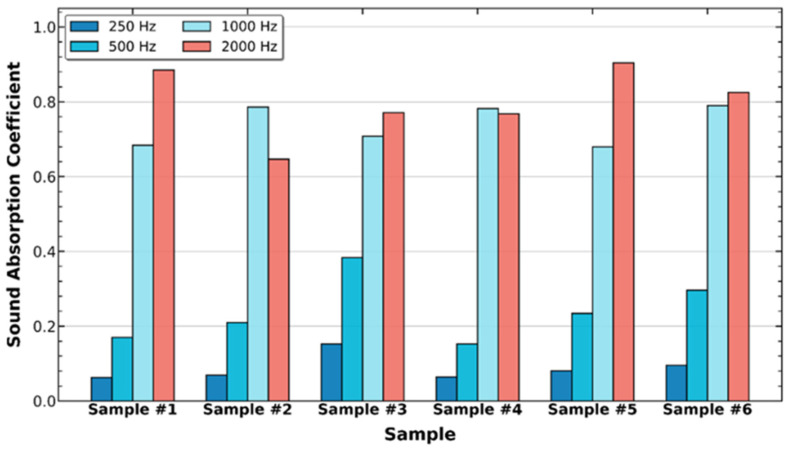
One-third octave values of SAC at 250, 500, 1000, and 2000 Hz.

**Figure 8:**
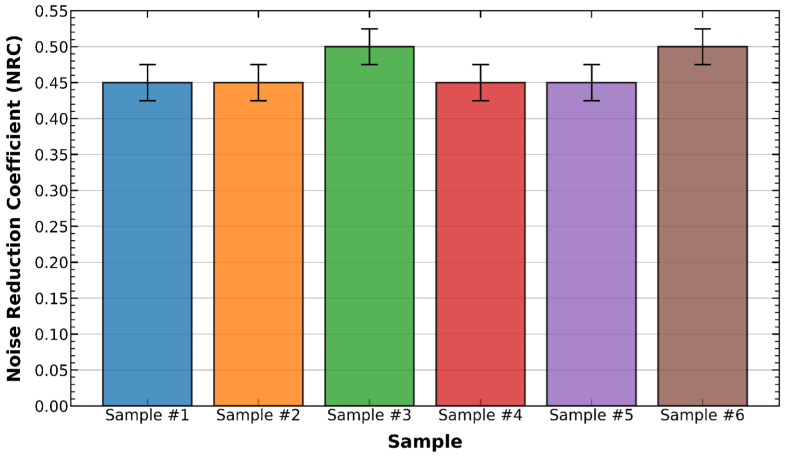
Noise reduction coefficient of the composites. The absolute uncertainty of the error bar is ±0.025 for all samples.

The promising sound absorption coefficients (SAC) are attributed to the air passages present within the composite samples. These passages enhanced the material’s absorptivity, thereby improving the overall SAC, as will be shown by the surface morphology of the composites of MIG + DPSF and CFP + DPSF. It is worth mentioning that the SAC is highly affected by the density, thickness, porosity, and percent of polymerized binders in the composites. Therefore, the incident sound waves are dissipated by viscous and thermal losses inside the inter-fiber air channels—as long as those channels are not continuous through the thickness of the composite boards—and hence enhance the SAC. A comparison between samples 1, 2, and 3, indicates that as the percent of DPSF (fibrous porous skeleton) decreases in the composite, it leads to enhancements in the absorption at a lower frequency. It is true, as mentioned earlier, that the SAC is >0.4 at frequencies of 500, 630, and 800 Hz for samples 3, 2, and 1, respectively. However, at mid-to-high frequencies ≥1000 Hz, the SAC is >0.68 for samples 1 and 2. The same observation is true for the composite samples 4, 5, and 6 of CFP and DPSF. Figure 7 shows a bar chart of the one-third octave band SAC values measured at 250, 500, 1000, and 2000 Hz. The noise reduction coefficient (NRC) is calculated from these measurements and is presented in Table 9 and Figure 8 as a bar chart, where the error bars present an uncertainty of ±0.025 for all samples. It should be noted that the absolute uncertainty for the NRC value is constant for all specimens, as it depends only on the assumed conservative value for the uncertainty in a single SAC measurement, which is adopted following Schultz et al. [32]: Δ (SAC) = ±0.05, and Δ (NRC) = 0.05/2 = ±0.025. Meanwhile, the relative uncertainty, which is defined as Δ (NRC)/(NRC) [39], depends on the magnitude of the NRC value itself, and it is in the range of 5.0% to 5.56%. Table 9 also compares the composites’ NRC with those of similar acoustic materials found in the literature. This comparison indicates that the NRC of the new composites is similar to or better than that of other materials. This highlights their potential for sound absorption in building construction, as they are cost-effective and have a lower environmental impact.

The high noise reduction coefficient (NRC) values, all greater than or equal to 0.45, further indicate the good sound absorption of the derived composites. These values are considered practical for real-world applications, consistent with previous findings [44,45,46].

**Figure 9:**
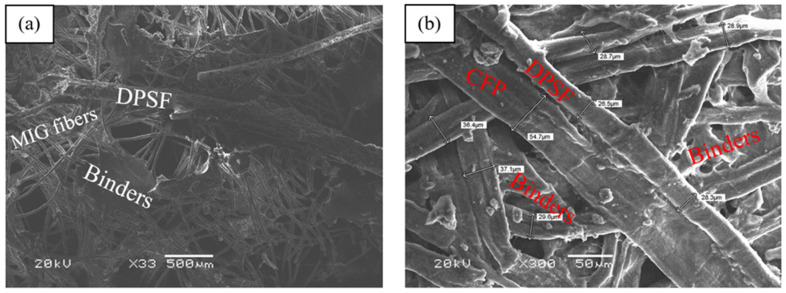
Surface morphology of the composites: (**a**) MIG and DPSF sample #2 and (**b**) CFP and DPSF sample #5.

Figure 9 presents samples of the surface morphology of the composites: sample #2 (MIG + DPSF) in Figure 9a and sample #5 (CFP + DPSF) in Figure 9b. The magnifications of ×33 and ×300, indicated by the scale bars in Figure 9 for sample 2 (Figure 9a) and sample 5 (Figure 9b), respectively, reveal both the inter-fiber architecture and the binder/fiber interface. The MIG and DPSF composite sample indicates that the MIG fibers’ diameter are smaller than that of the DPSF, as shown in Figure 9a. The CFP fibers are wider (flatter) than the DPSF, as shown in Figure 9b. In both figures, the binder holds the fibers together to form a more compact composite. It should be noted that small voids in the figures lead to a reduced thermal conductivity coefficient, as shown in Figure 5.

**Figure 10:**
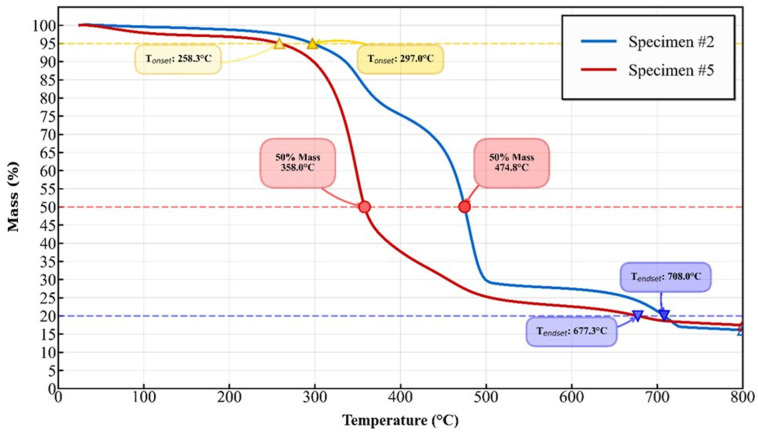
Thermogravimetric analysis (TGA) of the composites showing their decomposition.

**Figure 11:**
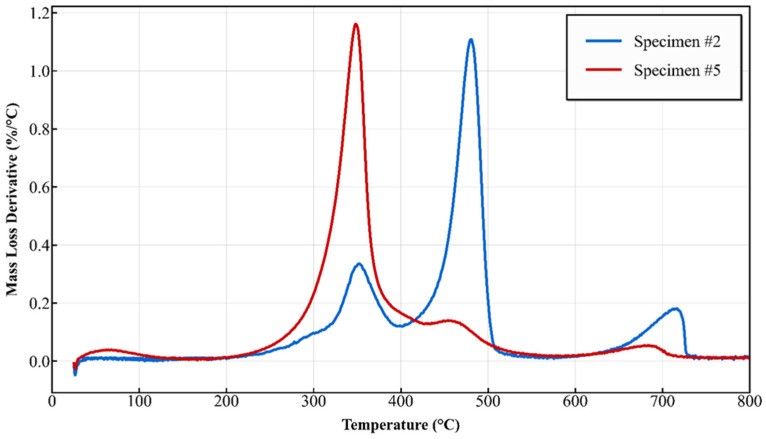
Differential Thermogravimetric Analysis (DTGA) highlighting the rate of mass loss.

Figure 10 presents the thermogravimetric analysis of the composites number 2 (MIG + DPSF) and 5 (CFP + DPSF). This figure shows that both composites are thermally stable up to 297 °C and 258.3 °C for samples numbers 2 and 5, respectively, where the composites lose their water vapor at this stage, corresponding to only 5% of their mass. The second degradation stage occurs where the composites lose 50% of their mass, specifically at 474.8 °C and 358 °C for numbers 2 and 5, respectively. The third stage is specified by an 80% mass loss at temperatures of 708.0 °C and 677.3 °C for numbers 2 and 5, respectively. Both composites reach char at 800 °C with approximately 15% leftover mass. Figure 11 shows the Differential Thermogravimetric Analysis (DTGA), where the peaks indicate the temperature of the maximum degradation rate for that specific stage, which is compatible with those of Figure 10. This figure confirms that these composites can be reliably used for thermal insulation, as they are thermally stable up to high temperatures.

**Figure 12:**
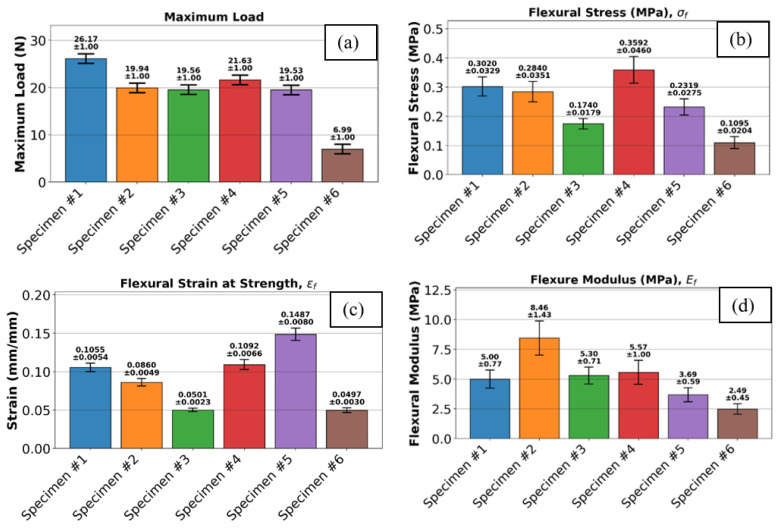
Mechanical properties of the composites: (**a**) maximum load, (**b**) flexural stress *σ_f_*, (**c**) flexural strain *ϵ_f_*, and (**d**) flexural modulus E_*f*_.

Figure 12 shows the results of the bending test’s mechanical properties, such as the maximum load (Figure 12a); the maximum flexural stress *σ_f_* attained, which indicates the material’s resistance to bending per unit area (Figure 12b); the flexural strain ϵ*_f_* at the maximum flexural stress, representing the physical deformation caused by flexural stress (Figure 12c); and the flexural modulus *E_f_*, which is the ratio of flexural stress to flexural strain within the material’s elastic region (Figure 12d). It should be noted that among the first three composites of MIG and DPSF, sample number two has the highest flexural modulus, and among the other three composites of CFP and DPSF, number four has the highest. It should be mentioned that the density, the ratio of the polymerized binder, and the thickness of the composites play a vital role in the flexural properties, which control the coherence of the composite and then the degree to withstand the load and the resistance to deformation. The mechanical properties of the table format, load deflection, and stress/strain profiles of the composites can be found in the Supplementary File.

**Figure 13:**
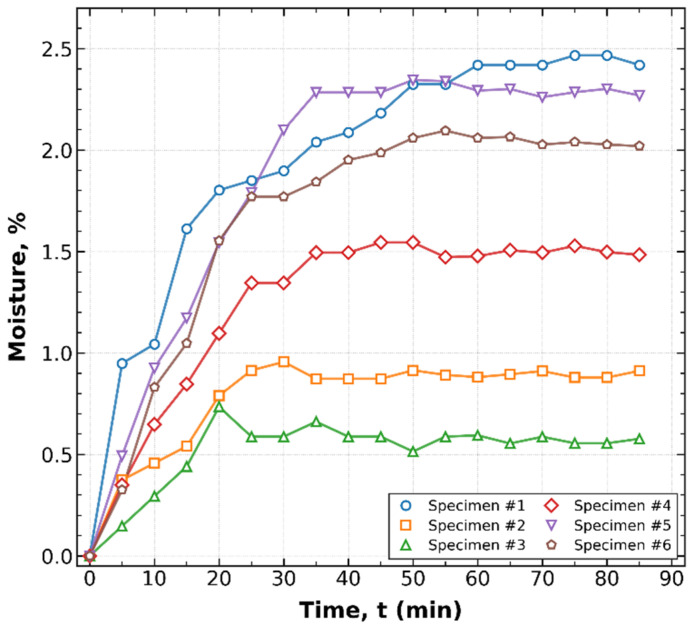
Moisture profiles for the composite samples.

Figure 13 presents the moisture content profiles for the MIG + DPSF composite specimens (samples 1, 2, and 3) up to their steady state. The results indicate that higher DPSF percentages increase moisture absorption. This trend is expected because bio DPSF is more hydrophilic and absorbs more moisture than MIG, which consists of recycled non-woven polypropylene. Consequently, samples 1 (65.2% DPSF), 2 (45.9% DPSF), and 3 (19.4% DPSF) exhibit steady-state moisture contents of approximately 2.5%, 0.9%, and 0.6%, respectively. Conversely, the remaining three composites (samples 4, 5, and 6) consist primarily of highly hydrophilic natural plant cellulose. This composition induces competition between the DPSF and CFP for moisture absorption; however, increasing the polymerized binder content restricts this competition. Consequently, when the binder ratio remains relatively constant, increasing the CFP percentage drives higher moisture absorption. This dominant effect is evidenced by the moisture content rising from 1.5% in sample 4 (23.2% CFP) to 2.3% in sample 5 (46% CFP). Furthermore, while sample 6 was expected to exhibit a higher moisture content than sample 5, the elevated percentage of polymerized binder likely suppressed its hydrophilic behavior, limiting its moisture absorption to approximately 2%. Nevertheless, these composites have a low moisture content, which aligns with Bainbridge’s recommended limits for thermal insulation [47].

## 5. Conclusions

New composite materials, developed from date palm surface fibers hybridized with either discarded medical isolation gowns or waste coffee filter papers, show promise for thermal insulation and sound absorption in building construction as sustainable alternatives to crude oil-based materials. This conclusion is based on a minimum thermal conductivity coefficient ranging from 0.0493 to 0.052 W/(m·K) at a room temperature of approximately 24 °C; the maximum ones are 0.0613 to 0.065 W/(m·K) at about 82 °C. Additionally, the sound absorption coefficient is ≥0.4 at frequencies of 500 Hz or higher, and the noise reduction coefficient is ≥0.45. The composites also exhibit a solid flexural modulus between 2.49 and 5.57 MPa. Thermally, the coffee filter paper hybrids remain stable up to 258.3 °C, while the medical isolation gown hybrids are stable up to 297.0 °C. Furthermore, the developed composites exhibited a moisture content ranging from 0.51% to 2.50%, which falls within the acceptable limit for thermal insulation materials.

## 6. Limitations and Future Work

While this present study demonstrates the technical feasibility of converting three abundant waste streams (DPSF, CFP, MIG) into functional partially bio-based insulation composites with thermal and acoustic performance comparable to commercial unconventional insulators, several limitations should be acknowledged: (i) Scalability. The composites were prepared at a laboratory scale (30 × 30 cm^2^ mold) using manual mixing and a hydraulic press. Industrial-scale production using continuous pressing would require optimization of fiber/binder distribution, energy consumption, and process throughput, as well as sourcing logistics for the three waste streams. (ii) Durability and Weatherability. Long-term aging under cyclic temperature, humidity, UV radiation, and biological attack (mold, fungi) was not investigated. The cellulosic fractions are inherently hydrophilic, and PVA may undergo gradual deacetylation and yellowing under UV exposure. (iii) Fire Performance. Standardized fire-reaction testing was not conducted; the TGA results, although encouraging, do not constitute fire-code compliance. (iv) Antimicrobial Safety. Dedicated antimicrobial activity testing was not performed. (v) Lifecycle and Economic Assessment. A full lifecycle assessment (LCA) and cost analysis comparing the present partially bio-based composites with conventional EPS, XPS, glass wool, and rock wool was beyond the scope of this study and is required to fully quantify the environmental and economic benefits. (vi) Field Validation. The thermal and acoustic measurements were obtained under controlled laboratory conditions (HFM 436, impedance tube). In situ measurements in actual building envelopes—using guarded hot-box and reverberation-room methods, and including hygrothermal monitoring—will be necessary to validate field performance. Future work will address these limitations through (a) pilot-scale manufacturing trials, (b) accelerated aging campaigns, (c) full fire characterization with environmentally compatible flame retardants, (d) antimicrobial functionalization, (e) lifecycle and cost analysis, and (f) in situ field validation in a demonstrator wall assembly.

## Figures and Tables

**Table 1:** Prepared hybrid composite samples using wood adhesive (WA) as a binder.

Material	Hybrid Composites					
	MIG + DPSF #1	MIG + DPSF #2	MIG + DPSF #3	CFP + DPSF #4	CFP + DPSF #5	CFP + DPSF #6
Gown (MIG), %	21.8	45.9	58.3	0.0	0.0	0.0
Coffee Filter (CFP), %	0.0	0.0	0.0	23.2	46	65.6
Date palm surface fibers (DPSF), %	65.2	45.9	19.4	69.4	46	21.9
The ratio of the polymerized binder to the total mass %	13	8.2	22.3	7.4	8.0	12.5
Thickness (mm)	20	18.0	23.0	17.0	19.0	17.0
The volume of the sample (cm^3^)	1800	1620	2070	1530	1710	1530
Density of dried samples (kg/m^3^)	223.3	235.2	217.4	247.0	222.5	261.4
Total dried mass (g)	402	381	450	378	380.4	400.0

**Table 2:** Initial preparation of dry hybrid composites.

Material	Dry Hybrid Composites (No Binders)					
	MIG + DPSF #1	MIG + DPSF #2	MIG + DPSF #3	CFP + DPSF #4	CFP + DPSF #5	CFP + DPSF #6
Gown (MIG), %	25%	50%	75%	0.0	0.0	0.0
Coffee Filter (CFP), %	0.0	0.0	0.0	25%	50%	75%
Date palm surface fibers (DPSF), %	75%	50%	25%	75%	50%	25%

**Table 3:** Flexure specimens’ dimensions.

Specimen ID	Thickness d (mm) ± 1.0 mm	Width b (mm) ± 1.0 mm	Support Span L (mm) ± 1.0 mm
#1 (MIG + DPSF)	20	52	160
#2 (MIG + DPSF)	18	52	160
#3 (MIG + DPSF)	23	51	160
#4 (CFP + DPSF)	17	50	160
#5 (CFP + DPSF)	19	56	160
#6 (CFP + DPSF)	17	53	160

**Table 4:** Uncertainties in flexural properties for all specimens.

Sample	Property	Original Value	Absolute Uncertainty	Percentage Uncertainty (%)
#1	Flexural Stress (σf)	0.3020 MPa	0.0329 MPa	10.89
	Flexural Strain (εf)	0.10547	0.005436	5.15
	Flexural Modulus (Ef)	4.9970 MPa	0.7651 MPa	15.31
#2	Flexural Stress (σf)	0.2840 MPa	0.0351 MPa	12.36
	Flexural Strain (εf)	0.08599	0.004897	5.69
	Flexural Modulus (Ef)	8.4620 MPa	1.4341 MPa	16.95
#3	Flexural Stress (σf)	0.1740 MPa	0.0179 MPa	10.29
	Flexural Strain (εf)	0.05013	0.002268	4.52
	Flexural Modulus (Ef)	5.2970 MPa	0.7102 MPa	13.41
#4	Flexural Stress (σf)	0.3592 MPa	0.0460 MPa	12.81
	Flexural Strain (εf)	0.10924	0.006569	6.01
	Flexural Modulus (Ef)	5.5750 MPa	0.9988 MPa	17.92
#5	Flexural Stress (σf)	0.2319 MPa	0.0275 MPa	11.86
	Flexural Strain (εf)	0.14873	0.008046	5.41
	Flexural Modulus (Ef)	3.6880 MPa	0.5925 MPa	16.07
#6	Flexural Stress (σf)	0.1095 MPa	0.0204 MPa	18.63
	Flexural Strain (εf)	0.04974	0.002991	6.01
	Flexural Modulus (Ef)	2.4920 MPa	0.4465 MPa	17.92

**Table 5:** Noise reduction coefficient’s uncertainty for all specimens.

Sample	NRC	Δ (NRC)	Relative Uncertainty (%)
#1 (MIG + DPSF)	0.45	±0.025	5.56
#2 (MIG + DPSF)	0.45	±0.025	5.56
#3 (MIG + DPSF)	0.50	±0.025	5.00
#4 (CFP + DPSF)	0.45	±0.025	5.56
#5 (CFP + DPSF)	0.45	±0.025	5.56
#6 (CFP + DPSF)	0.50	±0.025	5.00

**Table 6:** Uncertainty of the thermal conductivity coefficients at 25 °C and 80 °C.

Sample #	α at 25 °C	Δ α at 25 °C	α at 80 °C	Δ α at 80 °C	Δ α/α (%)
#1 (MIG + DPSF)	0.0500	±0.001275	0.0588	±0.001500	2.55
#2 (MIG + DPSF)	0.0513	±0.001313	0.0606	±0.001553	2.56
#3 (MIG + DPSF)	0.0503	±0.001276	0.0552	±0.001401	2.54
#4 (CFP + DPSF)	0.0528	±0.001355	0.0632	±0.001624	2.57
#5 (CFP + DPSF)	0.0543	±0.001387	0.0658	±0.001682	2.56
#6 (CFP + DPSF)	0.0543	±0.001394	0.0636	±0.001634	2.57

**Table 7:** Curve fitting constants and the coefficient of determination for each thermal conductivity profile.

Sample Number	A	B	R^2^, %
#1 (MIG + DPSF)	0.00016	0.046	98.8
#2 (MIG + DPSF)	0.00017	0.047	99.6
#3 (MIG + DPSF)	0.00009	0.048	95.1
#4 (CFP + DPSF)	0.00019	0.048	99.5
#5 (CFP + DPSF)	0.00021	0.049	91.6
#6 (CFP + DPSF)	0.00017	0.050	94.7

**Table 8:** Comparison between the current composites and both conventional and unconventional thermal insulation materials.

Materials	Density (kg/m^3^)	Thermal Conductivity Coefficient, α (W/(m·K))	Ref.
1 (DPSF + MIG)	223.3	0.049–0.059	This study
2 (DPSF + MIG)	235.2	0.051–0.061	This study
3 (DPSF + MIG)	217.4	0.050–0.056	This study
4 (DPSF + CFP)	247.0	0.052–0.064	This study
5 (DPSF + CFP)	222.5	0.052–0.065	This study
6 (DPSF + CFP)	261.4	0.053–0.063	This study
Hybrid of Eucalyptus Globulus leaves and wheat straw fibers	211.0	0.0460–0.0574	Ali et al. [33]
Hybrid (date palm tree surface fibers + Apple of Sodom fibers)	114.0–233.0	0.0423–0.0529	Alabdulkarem et al. [19]
Bagasse	70–350	0.0460–0.0550	Asdrubali et al. [34]
Straw bale	50–150	0.0380–0.0670	Asdrubali et al. [34]
Rice husk	154–168	0.0464–0.566	Asdrubali et al. [34]
Corn cob	171–334	0.101	Asdrubali et al. [34]
Kenaf	30–180	0.034–0.043	Asdrubali et al. [34]
Jute	26.1	0.0458	Korjenic et al. [35]
Flax	32.1	0.0429	Korjenic et al. [35]
Technical hemp	30.2	0.0486	Korjenic et al. [35]
Coconut fiber	40–90	0.0480–0.0576	Manohore et al. [36]
Recycled (PET)	15–60	0.034–0.039	Asdrubali et al. [34]
Recycled glass fiber	100–165	0.038–0.050	Asdrubali et al. [34]
Rock wool	40–200	0.033–0.040	Asdrubali et al. [34]
Polystyrene (Expanded, XPS)	15–35	0.031–0.038	Asdrubali et al. [34]
Polystyrene (Extruded, EPS)	32–40	0.032–0.037	Asdrubali et al. [34]
Sheep wool	10–25	0.038–0.054	Asdrubali et al. [34]
Recycled Polyethylene terephthalate (PET)	30	0.0355	Intini et al. [37]

**Table 9:** Noise reduction coefficient (NRC) of the composites.

Composite Number	Density, Kg/m^3^	Thickness (γ) (mm)	Frequency, Hz				NRC	Ref.
			250	500	1000	2000		
			SAC					
#1 (MIG + DPSF)	223.3	20	0.062	0.170	0.684	0.885	0.45	This study
#2 (MIG + DPSF)	235.2	18	0.069	0.210	0.786	0.647	0.45	This study
#3 (MIG + DPSF)	217.4	23	0.152	0.383	0.708	0.771	0.50	This study
#4 (CFP + DPSF)	247.0	17	0.064	0.152	0.782	0.768	0.45	This study
#5 (CFP + DPSF)	222.5	19	0.080	0.234	0.680	0.904	0.45	This study
#6 (CFP + DPSF)	261.4	17	0.095	0.296	0.790	0.825	0.50	This study
Polyurethane foam	95	NA	0.02	0.01	0.11	0.16	0.08	Martwong et al. [38]
Kenaf (light)	50	0.06	0.19	0.33	0.68	0.9	0.55	Berardi and Iannace [40]
Wood (fibers)	100	0.04	0.40	0.50	0.65	0.91	0.60	Berardi and Iannace [40]
Coconut	60	0.04/0.06	0.2	0.34	0.67	0.79	0.50	Berardi and Iannace [40]
Cork	100	0.03	0.02	0.10	0.30	0.86	0.30	Berardi and Iannace [40]
Cane (only wooden)	400	0.04	0.06	0.12	0.47	0.43	0.25	Berardi and Iannace [40]
Fleece (100% polyester) fiber	60	0.0035	0.08	0.12	0.19	0.21	0.15	Nandanwar et al. [41]
Queenscord fiber	160	0.0019	0.05	0.14	0.34	0.30	0.20	Nandanwar et al. [41]
Mesh fiber	100	0.0033	0.18	0.02	0.05	0.07	0.10	Nandanwar et al. [41]
Suede fiber	300	0.0006	0.09	0.13	0.24	0.28	0.20	Nandanwar et al. [41]
Wood fiberboard	480	0.018	0.11	0.14	0.21	0.34	0.20	Na et al. [42]
Palm oil leaves	152	0.010	---	0.05	0.08	0.19	0.10	Shahid et al. [43]
Lemongrass	201	0.010	---	0.06	0.15	0.45	0.20	Shahid et al. [43]

## Data Availability

The data sets used and/or analyzed during the current study are available from the corresponding author on reasonable request.

## Supplementary Materials

The following supporting information can be downloaded at: https://www.mdpi.com/article/10.3390/polym18111401/s1, Table S1: Mechanical properties of the hybrid composite samples; Figure S1: Load deflection profiles for the composite samples; Figure S2: Stress-strain profiles for the composite samples.
