# Supplementary material for: Characterization of Novel Partially Bio-Based, Waste-Derived Composites for Thermal and Acoustic Performance in Buildings
Source: Polymers (Basel). 2026 Jun 4;18(11):1401. doi: 10.3390/polym18111401 (PMC13259444; doi:10.3390/polym18111401)
Supplement: Supplementary file 1 [file polymers-18-01401-s001.zip › polymers-4294341-supplementary.pdf]

Table S1. Mechanical properties of the hybrid composite samples.

Specimen ID	Slope (N/ mm)	Flexure Modulus (MPa) , E_f	Flexural Stress (MPa), σ_f	Flexural strain at flexural strengt h, ϵ_f
**Specimen #1**	2.03	4.997	0.302	0.10547
**Specimen #2**	2.506	8.462	0.284	0.08599
**Specimen #3**	3.21	5.297	0.174	0.05013
**Specimen #4**	1.337	5.575	0.3592	0.10924
**Specimen #5**	1.383	3.688	0.2319	0.14873
**Specimen #6**	0.634	2.492	0.1095	0.04974

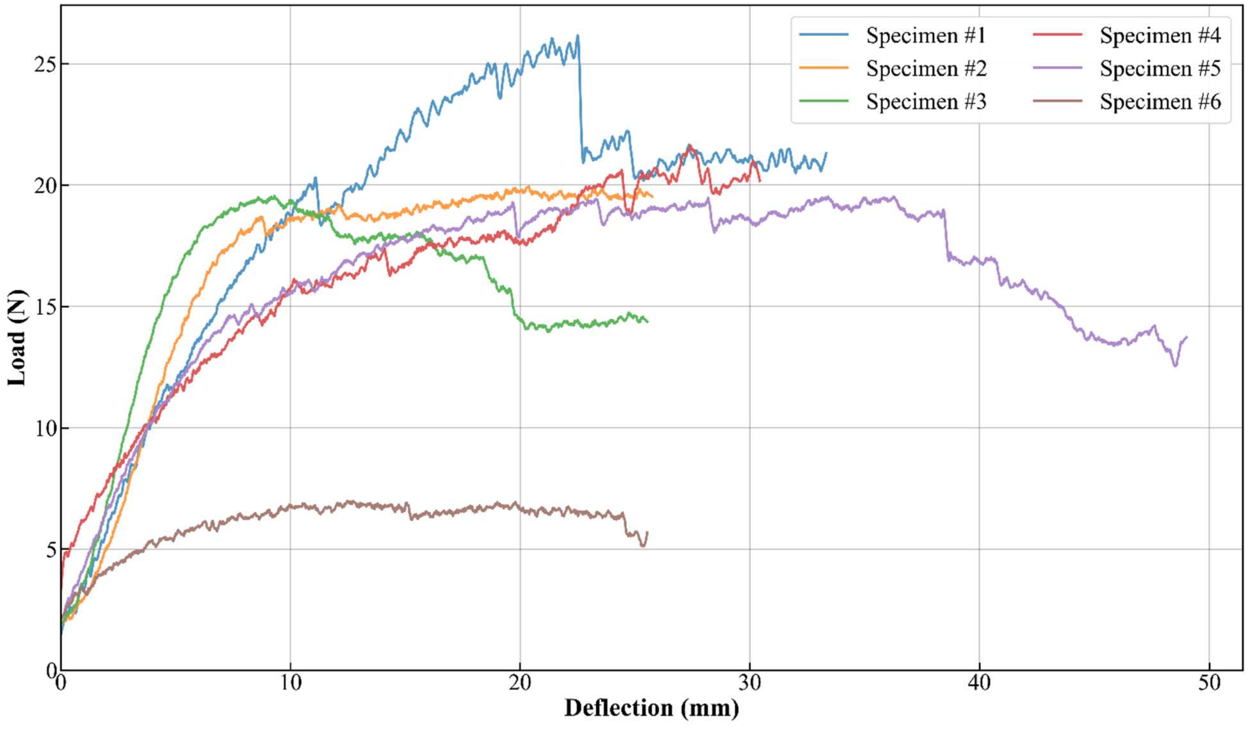

Figure S1. Load deflection profiles for the composite samples.

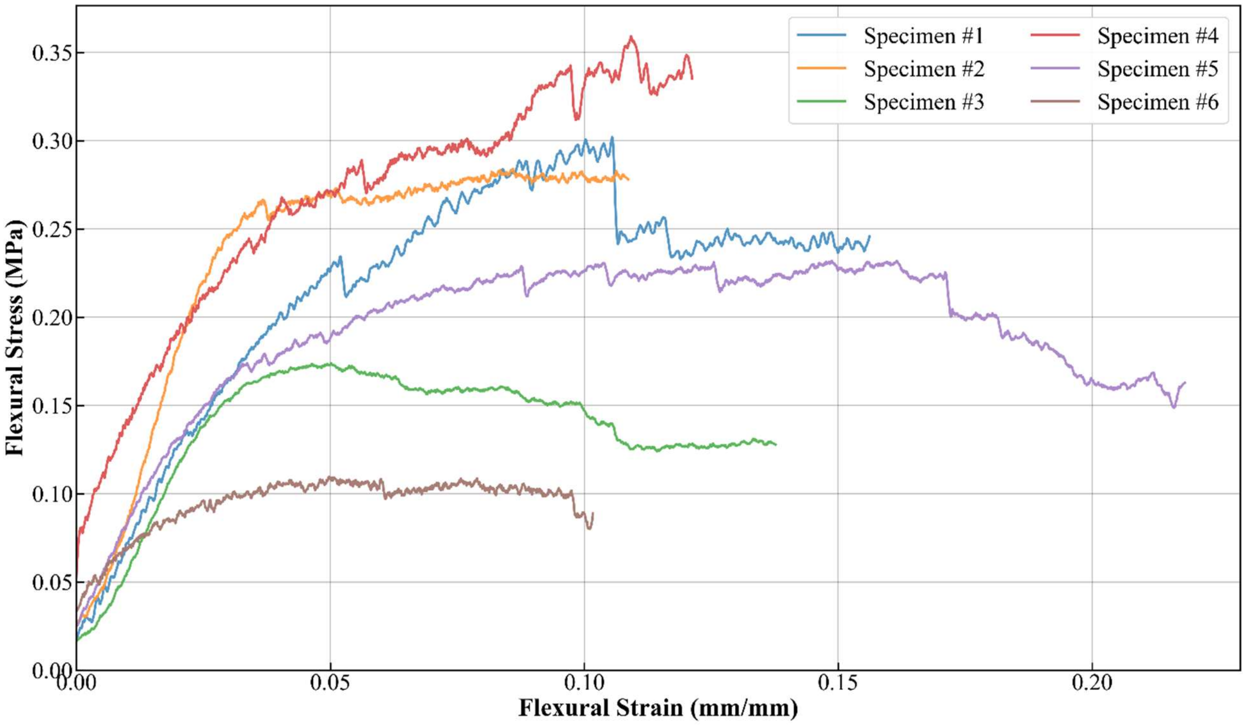

Figure S2. Stress-strain profiles for the composite samples.
